# The Use of Succinylcholine in Brugada Syndrome: A Case Report and Discussion of Literature

**DOI:** 10.1155/2019/5182672

**Published:** 2019-10-27

**Authors:** Marcus M. de Wolf, Nellie M. Rus, Guus H. Beljaars, Hanno L. Tan

**Affiliations:** ^1^Psychiatrist, GGZ Delfland, Centre for Mental Health, Delft, Netherlands; ^2^Anesthesiologist, GGZ Delfland, Center for Mental Health, Delft, Netherlands; ^3^Albert Schweitzer Hospital, Dordrecht, Netherlands; ^4^Cardiologist, Department of Cardiology, Academic Medical Center, University of Amsterdam, Amsterdam, Netherlands

## Abstract

We describe a patient with a major depression and a newly discovered Brugada syndrome, who was successfully treated with 35 electroconvulsive therapy sessions using succinylcholine as muscle relaxant. We discuss the use of succinylcholine in patients with Brugada syndrome.

## 1. Introduction

Brugada syndrome is an autosomal dominant inherited cardiac disease associated with increased risk of lethal cardiac arrhythmias and sudden cardiac arrest. It was first described by the brothers Brugada in 1992 [1]. The worldwide prevalence is estimated to vary between 5 and 20 per 10,000 individuals, but is significantly higher in some Asian and South-east Asian countries. In 10–20% of patients, a causative mutation in the *SCN5A* gene (which encodes the cardiac sodium channel) is found. Brugada syndrome is diagnosed when the distinctive coved-type ST elevation of at least 0.2 mV (type 1 ECG) occurs in at least one right precordial ECG lead (V1 or V2 or in leads in the 2^nd^ or 3^rd^ intercostal space cranially from V1 or V2), either spontaneously or after provocation with a class I antiarrhythmic drug (sodium channel blocking drug) [2]. A suspicion of Brugada syndrome may arise when these ECG leads exhibit coved-type ST elevation less than 0.2 mV or saddle-back ST elevation (type 2 or 3 ECG) [3].

Some medications and factors that influence the autonomic balance may evoke cardiac arrhythmias in patients with Brugada syndrome. In electroconvulsive therapy (ECT), multiple risk factors concur: the alternating stimulation of parasympathetic and sympathetic activity by the ECT-procedure itself, as well as the administration of several different types of medication because of the anesthesia. Thus far, literature provides only three reports on ECT in Brugada patients. Luckhaus et al. describe a patient with a several seconds lasting asystole after a single ECT session, in whom Brugada syndrome was discovered a year afterwards [4]. Tsutsumi et al. report about a patient with a Brugada ECG type 2 pattern who received 10 ECT sessions without complications [5]. Konishi et al. performed 8 uncomplicated ECT sessions in a patient with a Brugada type 1 pattern ECG [6]. Tsutsumi et al. as well as Konishi et al. preferred to use rocuronium (antagonized by sugammadex) for neuromuscular blockade instead of—the in ECT regularly used—succinylcholine. We describe a patient with Brugada syndrome who underwent 35 uncomplicated ECT sessions using succinylcholine as muscle relaxant. Written patient consent was obtained.

## 2. Case Presentation

A 50-year-old male patient was indicated for ECT treatment because of a severe depression with increasing suicidal thoughts, existing since two years. Several medication had been tried, (citalopram, escitalopram, nortriptyline, and lithium), but appeared not effective. His medical history included a hearing loss for which he had a hearing aid, but no other medical conditions.

On pre ECT examination, while on lithium and nortriptyline (no other medications), his ECG raised the suspicion of Brugada syndrome (type 2 ECG). There was no family history of sudden death at young age. Lithium and nortriptyline were discontinued because of their potential risk in Brugada syndrome. Cardiologic workup revealed no symptoms indicative of increased arrhythmia risk such as aborted cardiac arrest, (pre)syncope or palpitations, and cardiologic evaluation (exercise testing, 24-hour Holter recording, cardiac magnetic resonance imaging) was unremarkable. Based on these findings, it was concluded that his risk of potentially lethal arrhythmias was low, and that ECT-treatment was appropriate safe. Because of the severity of the depression and suicidality, it was decided not to postpone the treatment pending the further analysis of the suspected Brugada syndrome. After obtaining informed consent of the patient and his wife, which included information on possible cardiac risks, we started with dose titrated right unilateral ECT treatment, two times a week. The patient was anesthetized for each ECT with succinylcholine 80 mg, methohexital 90 mg and remifentanil 80 mcg, under the control of ECG, blood pressure, heart rate, pulse oximetry and electroencephalography.

**Figure 1:**
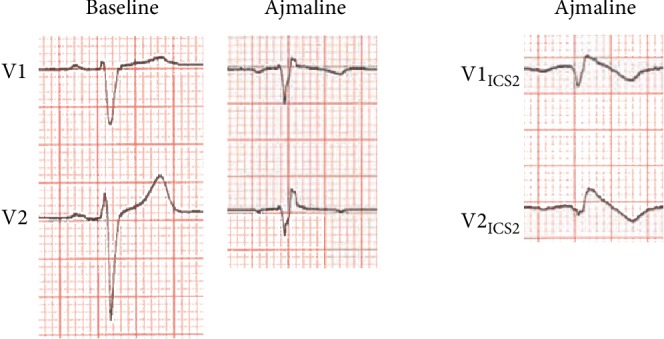
Provocation test with ajmaline elicited a type 1 ECG in the 2nd intercostal space cranially from V1 and V2 (V1_ICS2_ and V2_ICS2_).

During the ECT-course, further workup took place in an academic hospital. The suspicion of Brugada syndrome was confirmed when a provocation test with the class I antiarrhythmic drug ajmaline elicited a type 1 ECG (Figure 1). Genetic screening revealed no mutation in *SCN5A*. Yet, subsequent family screening yielded a positive ajmaline test in one brother and a negative test in the other brother, confirming Brugada syndrome in the patient.

The patient underwent subsequent ECT treatment without cardiac dysrhythmias or other medical problems. After thirteen ECT sessions, the depression was largely in remission. Because of the arrhythmia risk associated with the use of (tricyclic) antidepressants and lithium, we provided ECT as a maintenance treatment in a decreasing frequency. After a total of thirty-five sessions, we decided in consultation with the patient to stop the ECT treatment because of ECT-related cognitive complaints such as impairment of (biographical) memory. During the final weeks of ECT-treatment, venlafaxine 75 mg was started as maintenance treatment. Cardiologic investigations during the use of this drug (ECG, exercise testing, 24-hour Holter recording) revealed no indications for increased arrhythmia risk.

## 3. Discussion

There are some theoretical considerations against the use of succinylcholine in Brugada syndrome patients [5, 6]. First, succinylcholine is associated with bradycardia (especially in repeated gifts) and hyperkalemia. Furthermore, succinylcholine sporadically triggers malignant hyperthermia. In Brugada syndrome, this could be even more dangerous than in other patients.

On the other hand, the extent of bradycardia and hyperkalemia following a single dose of succinylcholine is generally mild and complications are rare. Also, the incidence of malignant hyperthermia is extremely low. General literature on anesthesia in Brugada syndrome patients does not advise against the use of succinylcholine [7–9]. Several case studies and series have been published in which succinylcholine was used without complications [7, 10, 11]. Therefore, succinylcholine is currently not categorized in the lists of medication that is to be (preferably) avoided, as provided by BrugadaDrugs.org, a website developed in collaboration with a panel of world-renowned experts on Brugada syndrome and broadly acknowledged in literature and guidelines [12]. Our case—describing only one patient, but nevertheless 35 uncomplicated anesthesia procedures—also illustrates that succinylcholine can be a safe option in Brugada syndrome patients.

The best available alternative for succinylcholine in ECT is the combination of the nondepolarizing agent rocuronium, followed by 8 mg/kg sugammadex at the end of the ECT treatment [13, 14]. This combination has been successfully used in two earlier described cases of ECT in Brugada syndrome patients [5, 6]. On the other hand, a recent case report suggested that sugammadex might have caused bradycardia and cardiac arrest in a previously healthy patient, which raises questions about its safety in Brugada syndrome [15]. Other disadvantages of the combination rocuronium/sugammadex are the high price, and probably the lack of availability of and experience with this medication in some ECT-facilities/countries.

In conclusion, when there are no specific contraindications like an increased risk of hyperthermia or hyperkaliemia, a single gift of succinylcholine can be considered a safe choice in ECT-patients with Brugada syndrome. The combination rocuronium/sugammadex seems a good alternative option.

## References

[1] Brugada P., Brugada J. (1992). Right bundle branch block, persistent ST segment elevation and sudden cardiac death: A distinct clinical and electrocardiographic syndrome: A multicenter report. *Journal of the American College of Cardiology*.

[2] Priori S. G., Wilde A. A., Horie M. (2013). Executive summary: HRS/EHRA/APHRS expert consensus statement on the diagnosis and management of patients with inherited primary arrhythmia syndromes. *EP Europace*.

[3] Wilde A. A., Antzelevitch C., Borggrefe M. (2002). Proposed diagnostic criteria for the Brugada syndrome: consensus report. *Circulation*.

[4] Luckhaus C., Hennersdorf M., Bell M., Agelink M., Zielasek W. J., Cordes J. (2008). Brugada syndrome as a potential cardiac risk factor during electroconvulsive therapy (ECT). *The World Journal of Biological Psychiatry*.

[5] Tsutsumi Y. M., Tomiyama Y., Horikawa Y. T. (2011). General anesthesia for electroconvulsive therapy with Brugada electrocardiograph pattern. *The Journal of Medical Investigation*.

[6] Konishi J., Suzuki T., Kondo Y., Baba M., Ogawa S. (2012). Rocuronium and sugammadex used effectively for electroconvulsive therapy in a patient with Brugada syndrome. *The Journal of ECT*.

[7] Sorajja D., Ramakrishna H., Poterack A. K., Shen W. K., Mookadam F. (2015). Brugada syndrome and its relevance in the perioperative period. *Annals of Cardiac Anaesthesia*.

[8] Kloesel B., Ackerman M., Sprung J. J., Narr B. J., Weingarten T. N. (2011). Anesthetic management of patients with Brugada syndrome: a case series and literature review. *Canadian Journal of Anesthesia*.

[9] Dendramis G., Paleologo C., Sgarito G. (2017). Anesthetic and perioperative management of patients with Brugada syndrome. *The American Journal of Cardiology*.

[10] Edge C. J., Blackman D. J., Gupta K., Sainsbury M. (2002). General anaesthesia in a patient with Brugada syndrome. *British Journal of Anaesthesia*.

[11] Vaccarella A., Vitale P., Presti C. A. (2008). General anaesthesia in a patient affected by Brugada syndrome. *Minerva Anestesiologica*.

[12] Postema P. G., Wolpert C., Amin A. S. (2009). Drugs and Brugada syndrome patients: review of the literature, recommendations, and an up-to-date website (www.brugadadrugs.org). *Heart Rhythm*.

[13] Hoshi H., Kadoi Y., Kamiyama J. (2011). Use of ocuronium-sugammadex an alternative to succinylcholine, as a muscle relaxant during electroconvulsive therapy. *Journal of Anesthesia*.

[14] Kadoi Y., Hoshi H., Nihida A., Saito S. (2011). Comparison of recovery times from rocuronium-induced muscle relaxation after reversal with three different doses of sugammadex and succinylcholine during electroconvulsive therapy. *Journal of Anesthesia*.

[15] Sanoja I. A., Toth K. S. (2019). Profound bradycardia and cardiac arrest after sugammadex administration in a previously healthy patient: a case report. *A & A Practice*.

